# Maximizing Roughness Factors in Oxide-Derived Copper Coatings through Electrodeposition Parameters for Enhanced Electrocatalytic Performance

**DOI:** 10.3390/nano13233064

**Published:** 2023-12-01

**Authors:** Eduard E. Levin, Alexander A. Kokin, Dmitriy A. Morozov, Victoria A. Nikitina

**Affiliations:** 1 Department of Chemistry, Lomonosov Moscow State University, 119991 Moscow, Russia; dmitrii.morozov@chemistry.msu.ru (D.A.M.); v.nikitina@skoltech.ru (V.A.N.); 2 Federal Scientific Research Centre “Crystallography and Photonics” of the Russian Academy of Sciences, 119333 Moscow, Russia; 3 Center for Energy Science and Technology, Skolkovo Institute of Science and Technology, 121205 Moscow, Russia; sashoknbv@mail.ru

**Keywords:** oxide-derived copper, Pb UPD, electrodeposition, real surface area

## Abstract

The pursuit of novel techniques for obtaining dispersed copper-based catalysts is crucial in addressing environmental issues like decarbonization. One method for producing nanostructured metals involves the reduction of their oxides, a technique that has found widespread use in CO_2_ electroreduction. Currently, the intrinsic activities of oxide-derived copper electrocatalysts produced via different routes cannot be compared effectively due to the lack of information on electrochemically active surface area values, despite the availability of electrochemical methods that enable estimation of surface roughness for highly dispersed copper coatings. In this study, we aim to explore the potential of oxide-derived copper to achieve a high electrochemically active surface area by examining samples obtained from acetic and lactic acid deposition solutions. Our results revealed that Cu_2_O oxides had distinct morphologies depending on the electrodeposition solution used; acetate series samples were dense films with a columnar structure, while electrodeposition from lactic acid yielded a fine-grained, porous coating. The roughness factors of the electroreduced films followed linear relationships with the deposition charge, with significantly different slopes between the two solutions. Notably, a high roughness factor of 650 was achieved for samples deposited from lactic acid solution, which represents one of the highest estimates of electrochemically active surface area for oxide-derived copper catalysts. Our results highlight the importance of controlling the microstructure of the electrodeposited oxide electrocatalysts to maximize surface roughness.

## 1. Introduction

One of the strategies to mitigate anthropogenic carbon dioxide emissions is the electroreduction of CO_2_ using sustainable energy, such as solar and wind power, to value-added chemicals, such as syngas, formate, C2 and C3 products, which can potentially enable a carbon-neutral energy cycle [1,2,3]. Copper-based materials represent the only category of electrocatalysts reported to date that can reduce CO_2_ to hydrocarbons and alcohols, while nanostructuring strategy enables the tuning of the selectivity towards multi-carbon products [4,5]. One method used to produce nanostructured metals involves the reduction of their oxides, a technique that has found widespread use in CO_2_ electroreduction for the preparation of so-called oxide-derived copper [6,7,8,9,10], although hydroxides are also used as precursors in this process [11]. This approach is a relatively simple method of nanostructuring that can be performed electrochemically in a solution that is close or identical in chemical composition to the solution used for conducting the CO_2_ electroreduction reaction, with coulometric or amperometric control of the completeness of the oxide reduction. Electrodeposition, thermal annealing and the anodization of copper foils are the most common methods of obtaining copper oxides [12], which can then be converted to metallic copper prior to or during the electrochemical CO_2_ reduction experiment. Copper deposits obtained in this way should potentially have a high electrochemically active surface area (EASA), but reliable estimates of this value for copper films derived from deposited onto foils or formed by oxidation of the foils oxides are rare, despite the availability of electrochemical methods to assess the true surface area.

Since the literature currently lacks examples of systematic and comprehensive studies of the influence of oxide thickness and morphology on the resulting EASA and roughness factors (*R*_f_) of the copper deposits obtained after the oxide reduction, only fragmentary information is available. For instance, the *R*_f_ of a copper film produced after the reduction of electrodeposited from lactate solution, (pH~9) Cu_2_O reached the value of 56 after 40 min of oxide electrodeposition; however, no information on the thickness dependence of surface roughness was provided [13]. In Ref. [14], *R*_f_ values were much lower (10–15) for the reduced Cu_2_O, which was deposited from the lactic acid stabilized solution at pH 12. For the Cu_2_O electrodeposited from a lactic acid solution under strongly alkaline conditions with thicknesses varying from 0.1 to 8.8 µm, roughness factors in the range of 1–11 were reported [15]. The roughness factors of copper derived from the anodization or thermal treatment of copper foils vary in a wide range from 10 to 500 depending on the specific procedure for the electrode preparation [16,17]. Currently, there is a knowledge gap concerning the relation between the conditions for the copper oxide electrodeposition and the resulting EASA of the reduced copper catalyst. Our study aims to emphasize the importance of accurately estimating *R*_f_ values when the oxide-derived copper materials are applied as electrocatalysts, providing reliable values of surface roughness for copper coatings produced via the reduction of electrodeposited oxides as well as explore the thickness dependence of roughness factors for samples with different morphologies.

The estimation of surface area for electrochemically produced electrocatalysts is typically carried out by comparing the “double-layer” currents on nanostructured deposits with those on smooth copper foil, assuming a roughness factor of unity or with capacitance values in aqueous solutions [13,14,16,18]. However, measuring double-layer capacitance on highly dispersed nanoporous electrodes is unreliable and lacks reproducibility due to the limitations of the method and the complexity of the objects. The main sources of error are difficulty in finding a true double-layer region on polycrystalline copper in alkaline, carbonate or phosphate-buffered solutions due to anion adsorption in a wide range of potentials [19,20,21], as well as problems in measuring capacitive currents for nanoporous materials with a non-negligible uncompensated ohmic drop [22]. More reliable approaches using underpotential deposition of Pb or Tl [23,24,25] to estimate the electrochemically active surface area (ESCA) are less common [26].

In this work, we explore the potential of electrodeposited oxide-derived copper to achieve high surface roughness values. As objects for obtaining oxide-derived copper, we selected Cu_2_O electrodeposited using the two most commonly encountered solutions: acetate [27,28,29] and lactate [30,31,32]. Acetate solutions are known for producing large crystalline deposits [28,33], while lactate solutions offer a variety of structures, including dispersed ones [34,35]. Thus, these two deposition solutions provide objects with different morphologies, and the study of their influence on the EASA of the reduced oxides with the aim of obtaining high values of true surface area is the subject of this work.

## 2. Materials and Methods

### 2.1. Electrodeposition

The electrodeposition was performed in a glass three-electrode single-compartment cell in the potentiostatic mode at 60 °C. The temperature was kept constant using a digitally controlled water bath. A high-purity copper plate (99.999%) was used as a counter electrode. The reference electrode was an Ag/AgCl (3 M KCl) reference electrode. For long experiments, the reference electrode was placed in a salt bridge with a permeable ceramic membrane. For electrochemical and scanning electron microscopy (SEM) investigations, a copper foil of 99.96% purity (thickness of 0.25 mm and an area of 1 cm^2^) was used as the working electrode. The surface of the electrode was electrically isolated with PET tape so that the working area was about 1 cm^2^. Prior to electrodeposition, the working electrode surface was pretreated as follows: etched in hot H_2_SO_4_ solution (195 g·L^−1^, 60 °C) until the oxide film was removed (usually about 10–15 s), washed with water, dried and activated in another H_2_SO_4_ solution (17.5 g·L^−1^, room temperature) for 1–3 s. The described procedures in this and the following paragraph are derived from a Russian state standard for preparing surfaces for electroplating. The reported concentrations of solutions were chosen in our group after rigorous tests. The deposition charges *Q*_d_ were 1–17 C·cm^−2^.

For X-ray diffraction measurements, the deposition was carried out on a stainless-steel support. The surface isolation was performed in the same manner. Prior to electrodeposition, the surface was chemically degreased at 60 °C in a solution containing 15 g·L^−1^ NaOH, 35 g·L^−1^ Na_3_PO_4_·12H_2_O, 35 g·L^−1^ Na_2_CO_3_ and 5 g·L^−1^ Na_2_O(SiO_2_)_n_ for 20 min. Chemical degreasing was followed by electrochemical degreasing at 80 °C and current density 2 A·dm^−2^ in the solution of 40 g·L^−1^ Na_3_PO_4_·12H_2_O, 40 g·L^−1^ Na_2_CO_3_ anodically for 5 min and cathodically for 10 min. Activation was carried out in H_2_SO_4_ solution (100 g·L^−1^, room temperature) for 60 s. The deposition charge was 10 C·cm^−2^.

The solutions used for electrodeposition were:

1. For this experiment, 0.02 M Cu(OAc)_2_ was added to the acetate buffer solution with pH 4.8. The buffer solution was prepared by mixing 87.2 mL of 1 M CH_3_COOH and 50 mL of 1 M NaOH in 500 mL of water. After adding copper acetate, the pH of the solution was adjusted to the original buffer pH by adding a few drops of 1 M NaOH.
2. For this experiment, 0.02 M Cu(OAc)_2_ was added to acetate buffer solution with pH 5.5. The buffer solution was prepared by mixing 57.4 mL of 1 M CH_3_COOH and 50 mL of 1 M NaOH in 500 mL of water. After adding copper acetate, the pH of the solution was adjusted to the original buffer pH by adding a few drops of 1 M NaOH.
3. For this experiment, 0.4 M CuSO_4_, 3 M C_3_H_6_O_3_, aged for 48 h, pH 7.9. The pH of the solution was initially adjusted to 8.5, but upon ageing at 25 °C, the pH lowered to 7.9 and was not changed further. Ageing is mandatory because the dissolved species require no less than 24 h to reach equilibrium [36].

A deposition temperature of 60 °C was selected because, for both acetate and lactate solution, elevated temperature accelerates the process of electrocrystallization [27,37].

The electrodeposition was performed using Biologic SP-50 (BioLogic, Grenoble, France) and Multi PalmSens4 (PalmSens BV, Houten, The Netherlands) potentiostats.

### 2.2. Characterization

X-ray Diffraction (XRD) patterns of the deposits detached from stainless-steel substrates were collected using Bruker D8 Advance (Bruker Corporation, Billerica, MA, USA) diffractometer (Bragg–Brentano geometry, CuKα radiation, LynxEye detector). The deposits were scratched off the stainless-steel support and glued with a drop of a hair lacquer to a silicon zero-background holder. Full-profile calculations and phase quantitation were performed using the Rietveld method [38,39] with a derivative difference minimization routine implemented in DDM 1.95e software [40]. Scanning electron microscopy (SEM) images were obtained using FEI Scios (FEI Company, Hillsboro, OR, USA) (Schottky field emission gun, Everhart–Thornley detector (positive bias) and in-lens secondary electron detector, landing energy 1–5 kV) and JEOL JSM-6490 LV (JEOL, Ltd., Tokyo, Japan) (tungsten hairpin gun, Everhart–Thornley detector (positive or negative bias), accelerating voltage 30 kV) scanning electron microscopes. Cross-sections were prepared by cutting deposits on copper foil on a Leica EM TXP (Leica Microsystems GmbH, Wetziar, Germany) target preparation device, followed by Ar^+^ ion milling using the Hitachi IM4000Plus (Hitachi High-Technologies Corporation, Tokyo, Japan) ion milling system (acceleration voltage 5 kV, ion current 1 mA). The cross-section was taken through the center of the sample. Cross-sections images were obtained using the FEI Scios scanning electron microscope in two modes: electron-induced secondary electrons (SE) and in Ga^+^ ion-induced secondary electrons (iSE) at an operating voltage of 30 kV and a current of 50 pA.

### 2.3. Electrochemical Reduction and EASA Measurements

Prior to the UPD measurements, oxide deposits were pre-reduced in a solution of 0.1 M KOH by sweeping the potential from the open circuit value to −1.1 V vs. HgO/Hg (1 M NaOH) reference electrode and then holding this potential until the current dropped to background values.

Pb UPD measurements were performed in an electrochemical cell with reduced copper oxide as a working electrode, a graphite rod as a counter electrode, and 3 M AgCl/Ag as a reference electrode. The working electrode and counter electrode were in the same compartment (ca. 60 mL). The working electrode solution was deaerated prior to the measurements for 60 min, and argon flow was maintained above the solution during the measurements. Special care was taken to avoid the contact of the reduced copper oxide electrode with the aerated solution and at open circuit potential. To minimize the effect of diffusion limitations in the nanoporous films on the shape of the voltammograms, in some measurements the solution in the working electrode compartment was stirred with a magnetic stirrer at 400 rpm.

Perchloric acid (puriss. p.a., Sigma Aldrich, St. Louis, MO, USA), NaClO_4_ (>99.9%, Sigma Aldrich), PbO (>99.5%, Lenreaktiv, St Petersburg, Russia), HCl (puriss, p.a., Merck, Darmstadt, Germany), and NaCl (>99.8%, Reachim, Moscow, Russia) were used to prepare the Pb UPD solutions. The concentration of Pb^2+^ ions was 10 mM. A constant perchlorate concentration of 0.1 M was maintained, and the pH of the solution was adjusted to 3. Potential sweep was carried out at a rate 1 mV·s^−1^. The UPD charge was determined by integrating and then averaging the anodic and cathodic branches of the voltammograms. The specific charge value was assumed 310 µC·cm^−2^, which is the value to a close packed Pb monolayer [25,41]. The *R*_f_ value was calculated by dividing the charge value obtained by integrating the voltammogram by the scan rate and by the specific charge value of 310 µC·cm^−2^.

All the potentials are given vs. Ag/AgCl (3 M KCl). All the electrochemical measurements were performed using Biologic SP-50 and Multi PalmSens4 potentiostats.

## 3. Results and Discussion

### 3.1. Morphology and Composition of Electrodeposited Coatings

According to the literature, large crystals of Cu_2_O can be electrodeposited from buffered solutions with pHs between 4.8 and 5.8 and containing 0.02 M Cu(OAc)_2_ [27,28]. The deposition at 60 °C is about three times faster than at room temperature [27]. It was found, however, that the solution with pH 5.8 was unstable when heated: we observed a change in color and the formation of a white precipitate. For this reason, the pH was shifted from a value of 5.8 to lower values in steps of 0.1 until a stable solution was found at pH 5.5. Unlike the original paper [28], where the deposition potential was kept constant at different pH values, in this work, the deposition was carried out at constant overpotential for pH values of 4.8 and 5.5.

**Figure 1:**
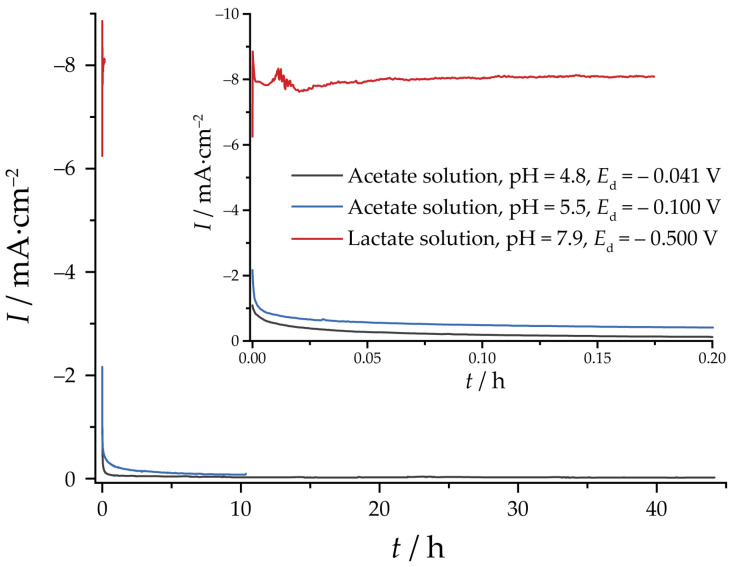
Current transients of the Cu_2_O electrodeposition from acetate and lactate solutions. The deposition charge is 10 C·cm^−2^.

Current transients at the conditions leading to Cu_2_O formation are collected in Figure 1 (see next paragraph for an explanation of the selection of deposition potentials). For the electrodeposition from acetate solutions, current transients usually reveal nucleation maxima [42,43]. However, in our case, the nucleation step was sufficiently fast so that these features overlap with the charging currents at short times. The current stabilizes on a timescale of minutes and remains almost unchanged further. For the lactate solution, similar current transients with a single nucleation maximum were reported earlier [44]. The overall current is significantly higher than for the specimens deposited from acetate solutions.

**Figure 2:**
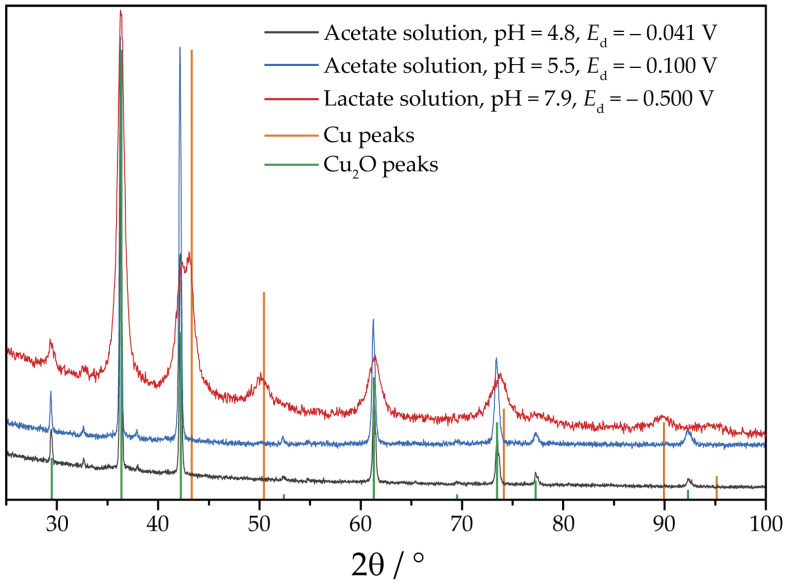
XRD patterns of the specimens selected for the electrochemical reduction. The deposition charge is 10 C·cm^−2^. Bars indicate positions and intensities of Cu (orange) [46] and Cu_2_O (green) [47]. The patterns are shifted along vertical axis for visual clarity.

**Figure 3:**
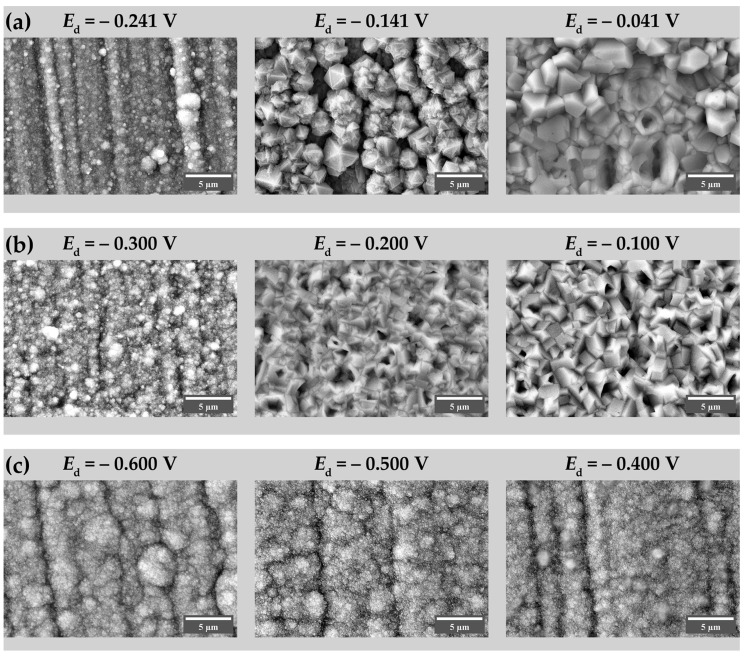
SEM images of the specimens: (**a**) acetate solution, pH 4.8, *Q*_d_ = 5 C·cm^−2^; (**b**) acetate solution, pH 5.5, *Q*_d_ = 5 C·cm^−2^; (**c**) lactate solution, pH 7.9, *Q*_d_ = 15 C·cm^−2^.

At pH 5.5, the redox potentials of Cu_2_O/Cu and Cu^2+^/Cu_2_O couples are 0.064 V and 0.218 V vs. Ag/AgCl (3 M KCl) reference electrode, respectively [45]. Electrodeposition was performed at potentials *E*_d_ = −0.100, −0.200 and −0.300 V, which corresponds to overpotentials of 318, 418 and 518 mV with respect to the Cu^2+^/Cu_2_O redox potential. XRD data indicate that the content of Cu_2_O naturally lowers with overpotential when Cu^2+^/Cu_2_O potential is increased (Figure S1, Table S1). At *E*_d_ = −0.100 V, the product is pure Cu_2_O (Figure 2). An attempt to deposit Cu_2_O from the solution with pH 4.8 revealed the difference in the acetate solutions. XRD analysis indicated that at *E*_d_ = −0.141 and −0.241 V (overpotentials 318 and 418 mV, respectively), the deposits are metallic copper with small (approximately 5 wt. %) amounts of Cu_2_O (Figure S2, Table S2); the latter is most likely a result of the oxidation of the deposits by the air. The only single-phased Cu_2_O was deposited at *E*_d_ = −0.041 V (overpotential 218 mV) (Figure 2). It could be noted that peak breadths on the corresponding XRD patterns (Figure 2) are low, indicating large coherently scattered domain sizes. The morphology of the specimens in the series is shown in Figure 3. Pure Cu_2_O deposits are composed of well-shaped micron-sized particles. At pH 4.8, the particle shape tends to be prismatic, while for pH 5.5, no particular crystal shape could be deduced. However, no porous structure was observed, which is reported in [27] for the electrodeposition under close solution composition and temperature, although some cavities are visible on the surface. The large particle size is in line with the narrow peaks observed on the XRD patterns.

The choice of the pH of the deposition solution and the values of the potentials for the lactate series are based on the information available in the literature on the dependence of the Cu_2_O particle size on both factors [37]. For a deposition potential sufficiently far from the co-precipitation potential of Cu and Cu_2_O, the particle size is the smallest at pH values between 8 and 10 [37,48]. Moreover, at these pH values, the crystallographic orientation axis is [100], whereas it switches to [111] at pH above 10 [37]. The [100] orientation is more favorable for the subsequent reduction, as the surfaces of the {100} family are less dense than those of the {111} family. For lactate solution, the deposits contain ~15 wt. % of Cu, and this quantity does not depend on the deposition potential (Figure S3, Table S3). Although the deposition of the Cu + Cu_2_O mixture is usually a feature of a galvanostatic deposition mode [49,50,51], it is possible that the mixture of phases could also be obtained in a potentiostatic regime [48]. The possible reason behind it is the shift of the near-electrode pH due to the hydroxylation of lactate, resulting in a deviation from the expected composition, as suggested in [52,53]. As compared to the peaks of the acetate series specimens, substantial peak broadening is visible on the XRD pattern (Figure 2). Its source is most likely the small size of the coherently scattering domain, being in the tens of nanometer range. This assumption is supported by SEM images. Morphologically, the obtained deposits do not differ significantly from each other (Figure 3). They are globular structures consisting of agglomerates with a diameter of about 2–3 μm composed of relatively spherical grains with a diameter of about 35–90 nanometers. The observed small grain size is thus responsible for the broadening of the XRD peaks (Figure 2).

A detailed investigation of the morphology of copper oxides deposited from lactate solutions at pH values in the range 8–10 and at lower/higher temperatures would be of interest for further studies, as different surface morphologies, potentially leading to higher surface roughness, could be formed under different deposition conditions.

### 3.2. Reduction of Oxides and Determination of EASA

**Figure 4:**
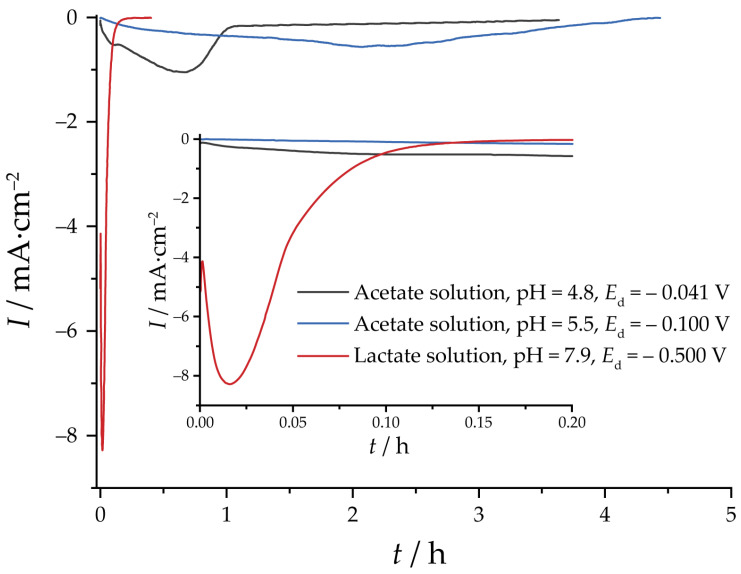
Current transients of electroreduction of the selected specimens with *Q*_d_ = 5 C·cm^−2^.

The reduction of Cu_2_O specimens carried out potentiostatically follows the same trends observed for electrodeposition. For the acetate specimens (Figure 4), the overall current is low. For pH 4.8, some features are present, resembling nucleation maxima. For the lactate specimen, the process was completed much faster, with clear current maxima. The differences in the rate of oxides reduction reflect the differences in the particle size, which is a limiting factor given the low electronic conductivity of Cu_2_O.

**Figure 5:**
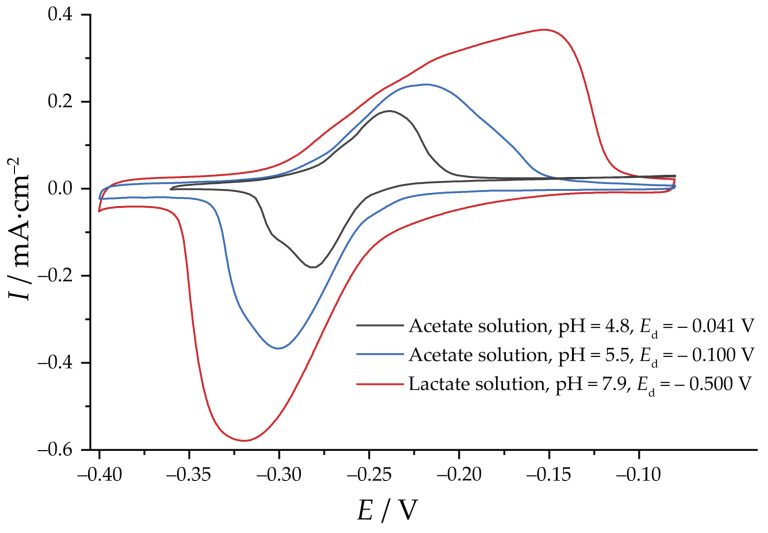
Cyclic voltammograms of the pre-reduced Cu_2_O specimens in the 10 mM Pb(ClO_4_)_2_ + 0.1 M NaClO_4_ + 1 mM NaCl solution, pH 3. Potential sweep rate is 1 mV·s^−1^. *Q*_d_ = 5 C·cm^−2^.

Figure 5 displays the cyclic voltammograms of the specimens in the Pb^2+^ ion solution, indicating variations in EASA, as reflected in the difference in total charges. To facilitate comparison across the different series, a *Q*_d_ value of 5 C·cm^−2^ was chosen as an arbitrary benchmark for depositing specimens within a reasonable time frame for all solutions examined in this study. At this deposition charge, the largest value of *R*_f_ for acetate solution at pH 4.8 was 34 for *E*_d_ = −0.041 V. For other values of *E*_d_, *R*_f_ does not exceed 5, which is in line with the phase composition of the deposits (Table S2). At pH 5.5, the value of *R*_f_ was 71 for *E*_d_ = −0.100 V. For *E*_d_ = −0.200 V and −0.300 V the determined values of *R*_f_ were 53 and 6, respectively, reflecting the decrease in Cu_2_O content (Table S1). For the lactate solution, no dependence of *R*_f_ on *E*_d_ was observed; therefore, for further comparison, *E*_d_ = −0.500 V was selected. At a deposition charge of 5 C·cm^−2^, the *R*_f_ was 205, which is notably larger than for the acetate series at pH 5.5. Because of the morphological similarity and impractically low deposition rate of Cu_2_O from the acetate solution at pH 4.8, further comparison between the series was undertaken for the deposits from acetate solution at pH 5.5 and the lactate solution.

**Figure 6:**
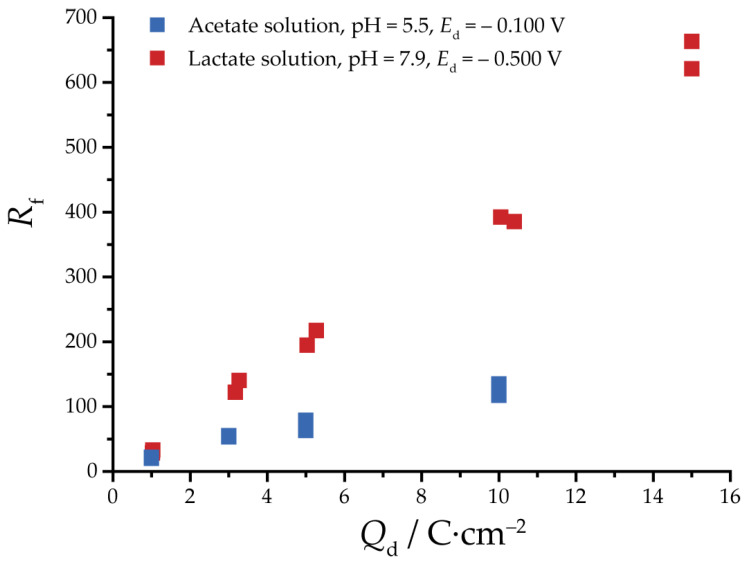
The plot of *R*_f_ vs. deposition charge for lactate and acetate (pH = 5.5) specimens.

For both solutions, a series of deposits were obtained for *Q*_d_ of 1, 3, 5 and 10 C·cm^−2^. To test reproducibility, *R*_f_ was determined twice for each deposition charge. The plots of *Q*_d_ vs. *R*_f_ are displayed in Figure 6. For both solutions, there is a linear relationship between *Q*_d_ and *R*_f_ at least up to charges of 10 C·cm^−2^. However, the slopes of these two dependencies are noticeably different. The dependence is quite flat for the acetate solution with pH 5.5, indicating that it is necessary to significantly increase the thickness of the sample to obtain a catalyst with a sufficiently high EASA after the Cu_2_O reduction. On the contrary, for lactate solution, this dependence is steeper. An additional specimen with *Q*_d_ = 15 C·cm^−2^ was obtained, for which a very high *R*_f_ = 650 was observed with no distortion of linear dependence of *Q*_d_ vs. *R*_f_. Although oxide-derived copper deposited from lactate solution can achieve a high roughness factor, selecting the optimal roughness of the electrocatalytic film requires a compromise between the film thickness and EASA. Thick nanoporous films may generate pH gradients due to diffusion limitations that can affect catalyst selectivity and activity.

### 3.3. Cross-Section Examination

A possible explanation for the different behavior of *R*_f_ vs. *Q*_d_ was sought in terms of morphological differences after the electroreduction of Cu_2_O. To identify morphological changes associated with the process, both surface and cross-sectional SEM images were examined.

**Figure 7:**
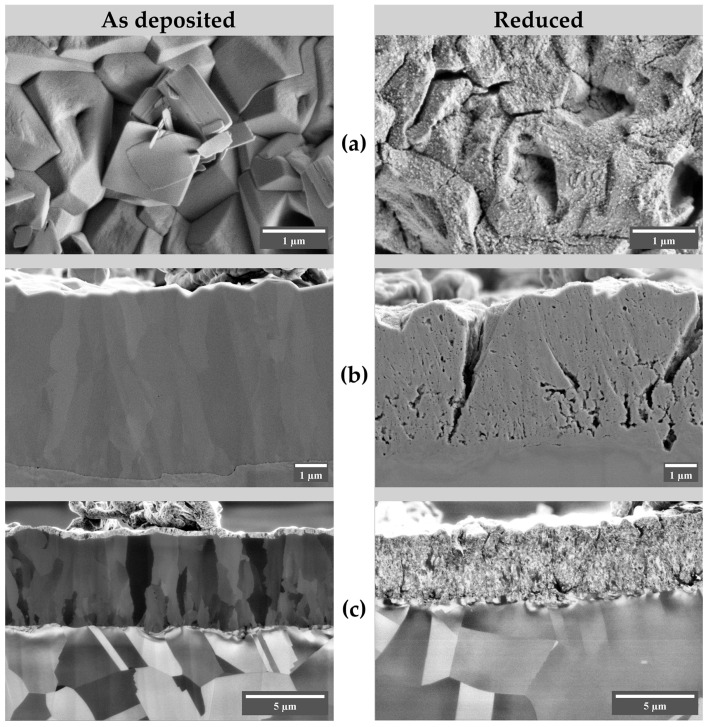
SEM images of the as-deposited and reduced specimens of the acetate series (pH 5.5, *E*_d_ = −0.100 V, deposition charge 5 C·cm^−2^). (**a**) surface morphology; (**b**) cross-section images, secondary electrons (SE); (**c**) cross-section images, ion-induced secondary electrons (iSE).

We found that the reduction induces notable changes in morphology observable both in surface morphology and in cross-sections (Figure 7 and Figure S4). The surface of the as-deposited film consists of highly intergrown micron-sized faceted particles. After reduction, at low magnifications, characteristic defects with circular cracking can be seen, which are absent in the microphotographs of the pristine samples (Figure S4). We assume that during the reduction of Cu_2_O, oxygen released on the surface of the deposit adjacent to the substrate forms gas bubbles, which, upon reaching a certain internal pressure, lead to local ruptures. Straight ruptures, indicative of large compression stresses, are observed in both acetate series samples (Figures S4 and S5), which are associated with a decrease in coating volume due to reduction.

The surface of the particles, initially smooth after the deposition, becomes noticeably rough due to the cracking and visible fragmentation of large crystallites into small particles (Figure 7). The cross-sectional images also show significant changes. Freshly deposited Cu_2_O at *E*_d_ = −0.100 V has a columnar structure with large grains and the height of the deposit, especially accentuated in the iSE mode; such a visualization regime offers improved contrast due to the channeling effect [54]. Upon reduction, the grains disintegrate, forming small particles. Electron-excitation images also show cracking of the sample throughout its thickness and the formation of pores and small particles. It is also possible to note a decrease in the height of the reduced deposit, which is likely associated with the difference in density between metallic copper and Cu_2_O. The fresh deposit has a height of approximately 5.7 μm. After the reduction, the height is reduced to 4.1 μm, 72% of the initial height, which is about the difference in terms of the densities of Cu_2_O and Cu (6.10 and 9.86 g·cm^−3^). This observation suggests that the deposit behaves like a dense, non-porous layer, exhibiting a tendency towards mechanical instability after reduction, which is associated with the appearance of a large number of cracks and ruptures.

**Figure 8:**
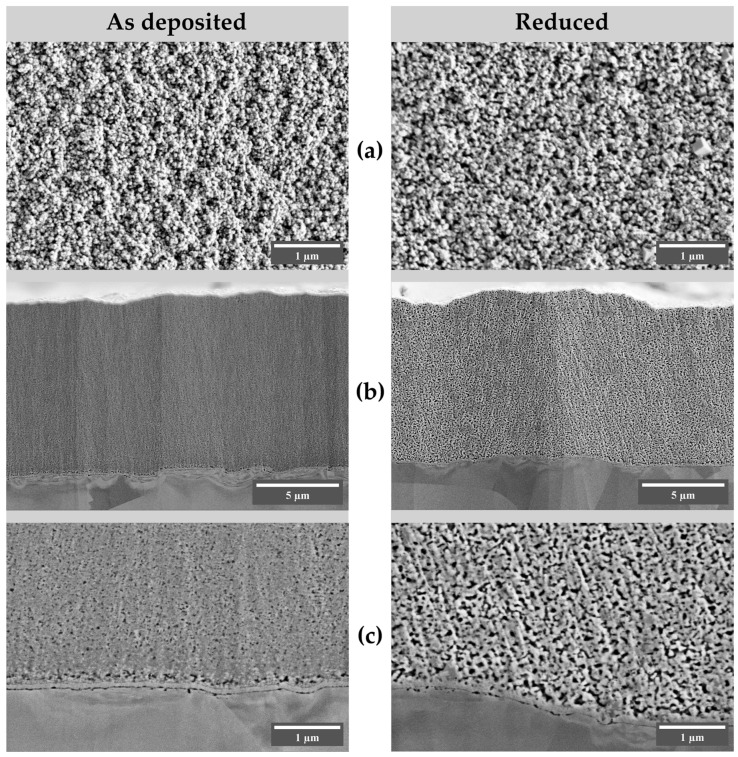
SEM images of the as deposited and reduced specimens of the lactate series (pH 7.9, *E*_d_ = −0.500 V, deposition charge 15 C·cm^−2^). (**a**) surface morphology; (**b**,**c**) cross-section images, secondary electrons (SE).

In the images of the cross-sections of the fresh and reduced samples of the lactate series, the following morphological features can be noted (Figure 8). The deposits have homogeneous porous structures, with the visible pore diameter being larger in the reduced sample. No circular cracking or tearing was observed for this specimen (Figure S6). In addition, there was no significant decrease in the height of the sample after reduction (10.4 μm after deposition, 10.0 μm after reduction), indicating that the sample is a porous, permeable structure capable of maintaining its external shape during the electroreduction process. As no separate copper layer was observed on the cross-section images, we can speculate that copper particles present in the coating form a three-dimensional non-compressible framework on which copper oxide grows, which may explain the increase in pore volume in the absence of noticeable isotropic compression, in contrast to that observed for the sample deposited from the acetate solution. Such porous structures should naturally feature a large specific surface area. This observation also serves as an explanation for the large currents during the reduction (Figure 4) of this specimen. Further studies are needed to investigate the restructuring of oxide-derived copper coatings under specific conditions of selected electrocatalytic processes, such as CO_2_ or nitrate reduction.

## 4. Conclusions

We conducted a comparative study on the EASA values of copper films obtained through the electroreduction of copper (I) oxides deposited from acetate and lactate solutions. Our findings indicate that reducing deposits with micron-sized, well-crystallized particles from the acetate series results in moderate roughness factors (not exceeding 125 for a deposition charge of 10 C·cm^−2^). On the other hand, reducing copper oxide deposited from lactate solution produces highly porous films with roughness factors up to 400 for a deposition charge of 10 C·cm^−2^. The roughness factors were found to depend linearly on the deposition charge. These results highlight the importance of controlling the microstructure of the deposits when selecting a catalyst preparation method and exemplify the possibility of obtaining copper-based electrocatalysts with the required EASA via tuning the parameters of copper oxide electrodeposition, such as pH, the deposition potential, and the deposition charge. Control of the nanoscale structure is key to ensuring a high specific surface area in the resulting oxide-derived copper and, in a broad sense, all oxide-derived metals. Since, in this work, we only examined the surface roughness of reduced copper oxide deposited from solutions with selected pH values at several overpotentials, it is expected that future studies will expand our knowledge of the influence of electrodeposition conditions on the resulting EASA values of copper-based electrocatalysts.

## Data Availability

The data presented in this study are available upon request from the corresponding author.

## Supplementary Materials

The following supporting information can be downloaded at: https://www.mdpi.com/article/10.3390/nano13233064/s1, Figure S1: XRD data for the specimens deposited from acetate solution with pH = 5.5; Table S1: XRD phase quantitation of acetate series specimens, pH = 5.5; Figure S2: XRD data for the specimens deposited from acetate solution with pH = 4.8; Table S2: XRD phase quantitation of acetate series specimens, pH = 4.8; Figure S3: XRD data for the specimens deposited from lactate solution with pH = 7.9; Table S3: XRD phase quantitation of lactate series specimens, pH = 7.9; Figure S4: SEM images of the specimens deposited from acetate solution (*E*_d_ = −0.100 V, *Q*_d_ = 5 C·cm^−2^) with pH = 5.5 after Cu_2_O reduction; Figure S5: SEM images of the specimens deposited from acetate solution (*E*_d_ = −0.041 V, *Q*_d_ = 5 C·cm^−2^) with pH = 4.8 after Cu_2_O reduction; Figure S6: SEM images of the specimens deposited from lactate solution (*E*_d_ = −0.500 V, *Q*_d_ = 15 C·cm^−2^) with pH = 7.9 after Cu_2_O reduction.
