# Supplementary material for: Maximizing Roughness Factors in Oxide-Derived Copper Coatings through Electrodeposition Parameters for Enhanced Electrocatalytic Performance
Source: Nanomaterials (Basel). 2023 Dec 1;13(23):3064. doi: 10.3390/nano13233064 (PMC10707879; doi:10.3390/nano13233064)

Supporting information

Maximizing Roughness Factors in Oxide-Derived Copper Coatings through Electrodeposition Parameters for Enhanced Electrocatalytic Performance

Eduard E. Levin[1,2,*], Alexander A. Kokin[3], Dmitriy A. Morozov[1,3] and Victoria A. Nikitina[1,3]

[1]Department of Chemistry, Lomonosov Moscow State University, 119991 Moscow, Russia
[2]Federal Scientific Research Centre "Crystallography and Photonics" of the Russian Academy of Sciences, 119333 Moscow, Russia

[3]Center for Energy Science and Technology, Skolkovo Institute of Science and Technology, 121205 Moscow, Russia

*Correspondence: levin@elch.chem.msu.ru

Figure S1. XRD data for the specimens deposited from acetate solution with pH = 5.5.

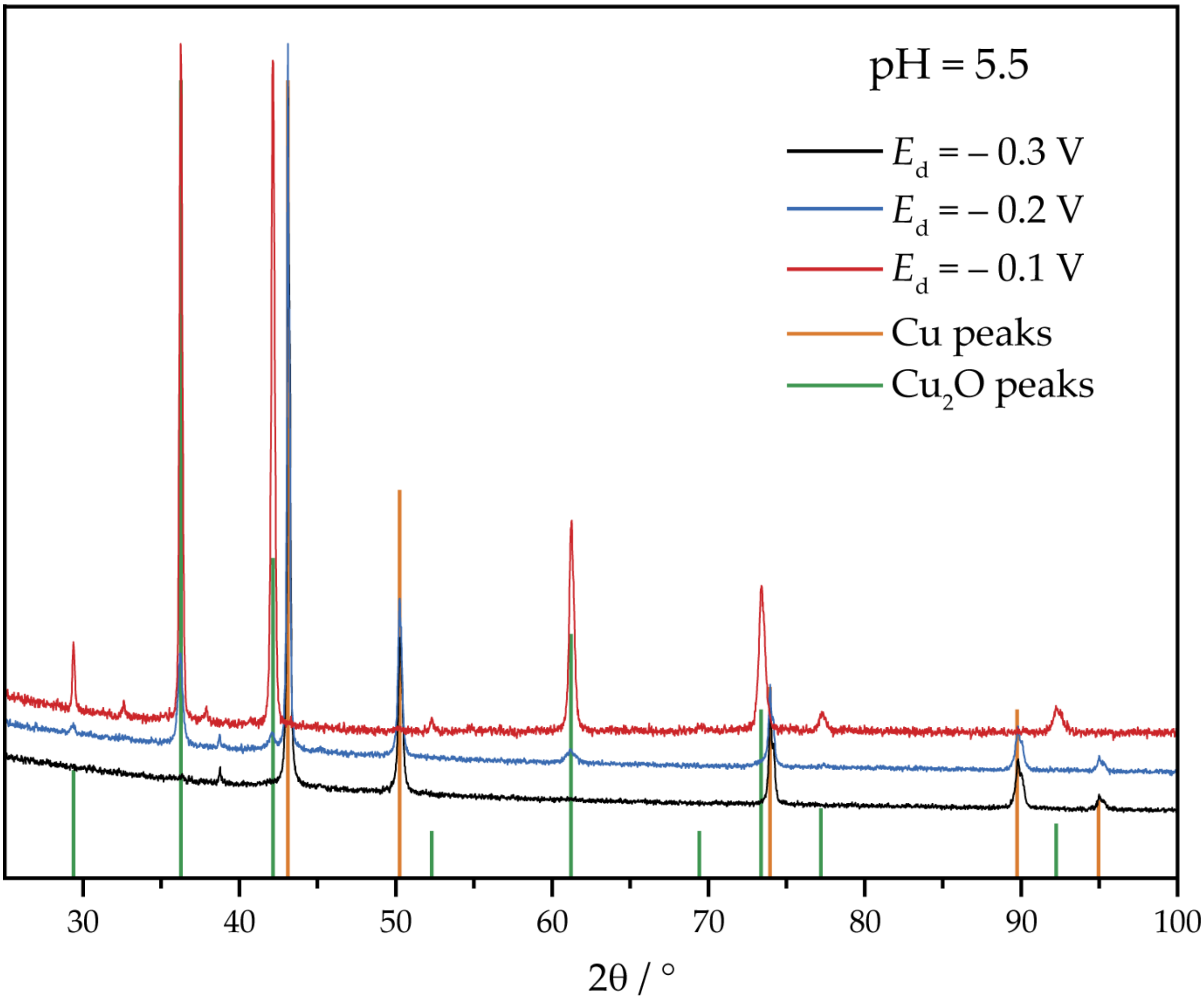

Table S1. XRD phase quantitation of acetate series specimens, pH = 5.5.

E_d, V	Cu, wt. %	Cu_2O, wt. %
–0.1	–	100
–0.2	83.6 ± 1.1	16.4 ± 1.0
–0.3	100	–

Figure S2. XRD data for the specimens deposited from acetate solution with pH = 4.8.

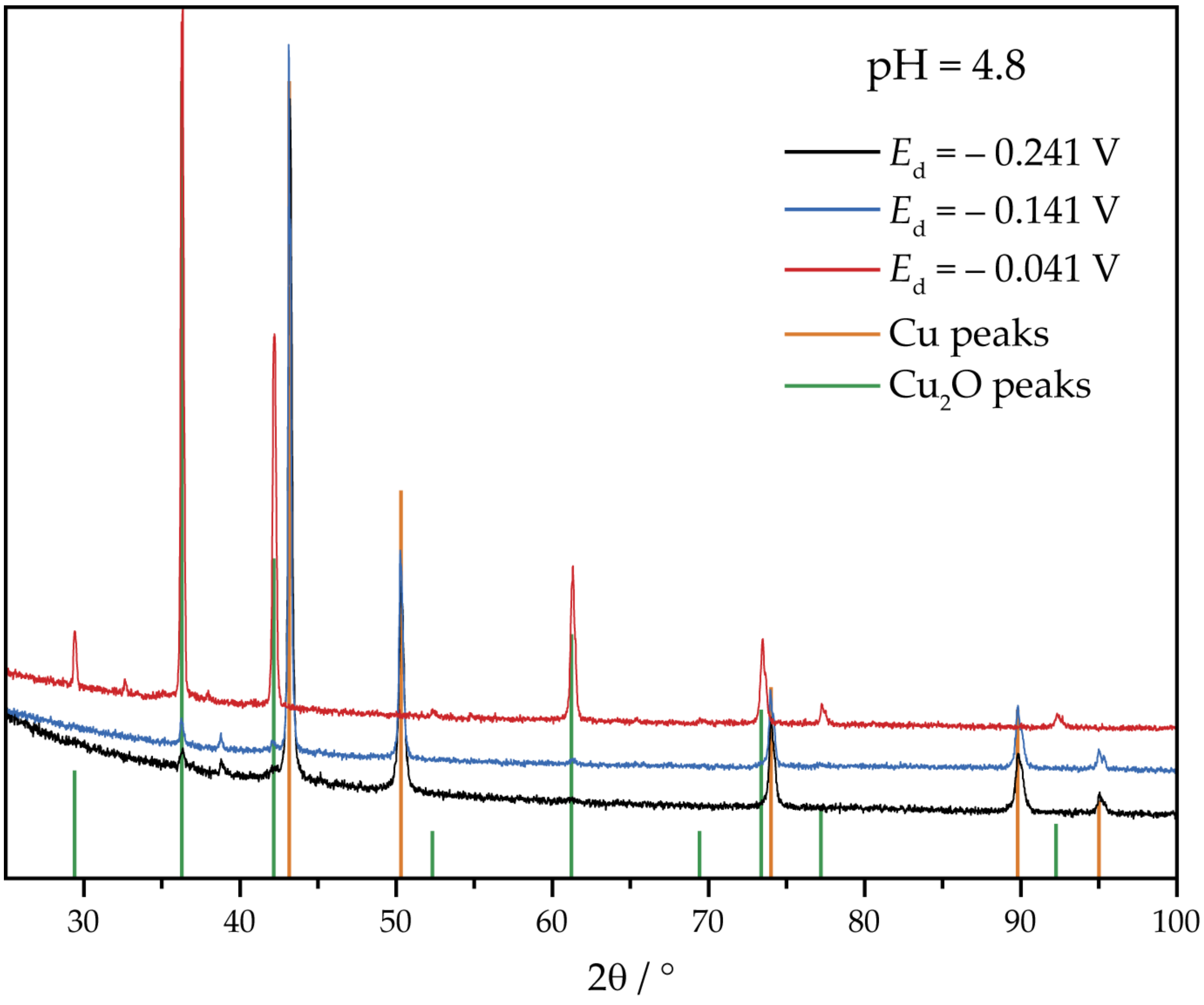

Table S2. XRD phase quantitation of acetate series specimens, pH = 4.8.

E_d, V	Cu, wt. %	Cu_2O, wt. %
–0.041	–	100
–0.141	94.7 ± 3.0	5.3 ± 0.3
–0.241	96.4 ± 2.0	3.6 ± 0.4

Figure S3. XRD data for the specimens deposited from lactate solution with pH = 7.9.

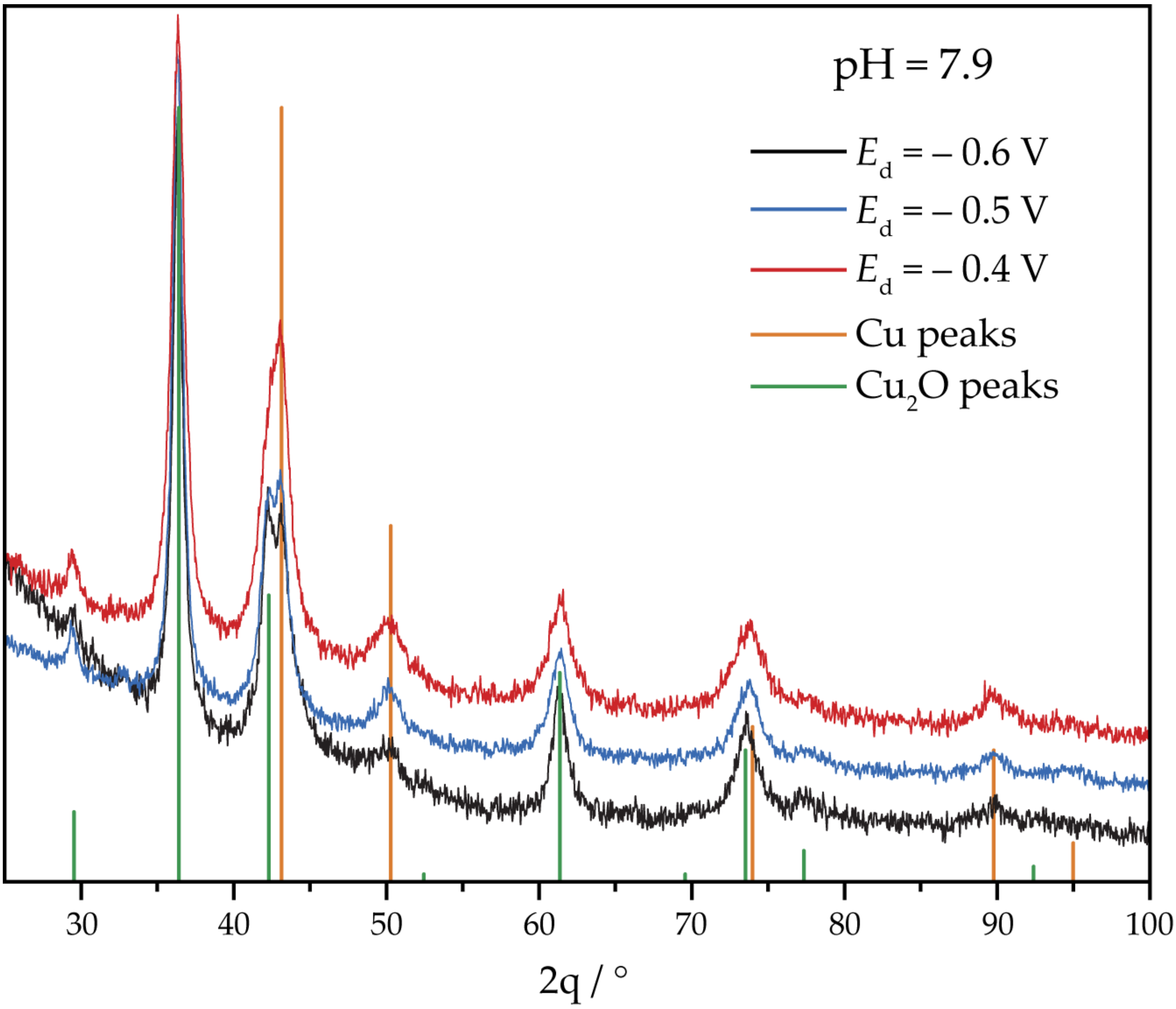

Table S3. XRD phase quantitation of lactate series specimens, pH = 7.9.

E_d, V	Cu, wt. %	Cu_2O, wt. %
–0.4	18.1 ± 1.4	81.9 ± 1.0
–0.5	16.6 ± 1.5	83.4 ± 1.0
–0.6	21.2 ± 2.0	78.8 ± 3.3

Figure S4. SEM images of the specimens deposited from acetate solution (E_d = –0.100 V, Q_d = 5 C·cm^{-2}) with pH = 5.5 after Cu_2O reduction.

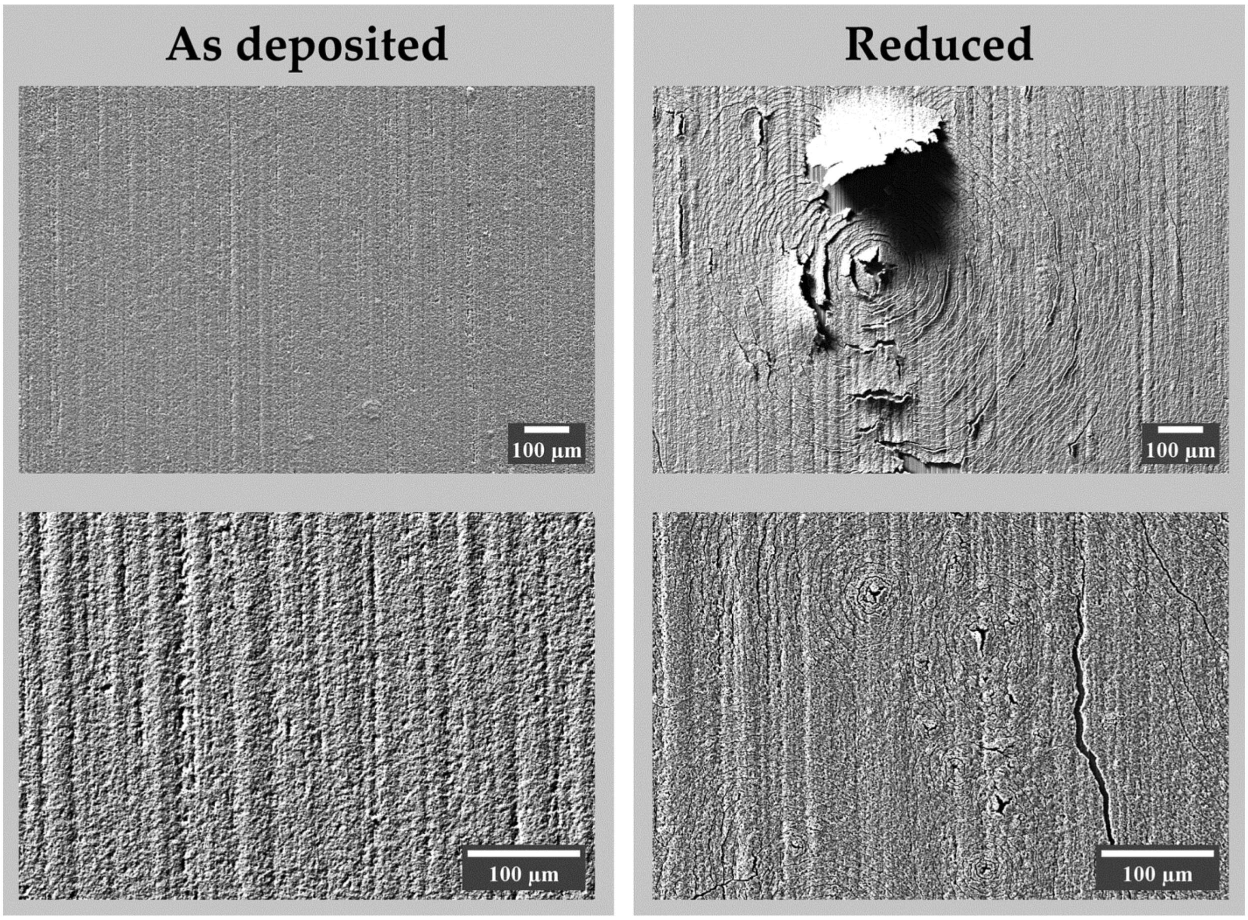

Figure S5. SEM images of the specimens deposited from acetate solution (E_d = –0.041 V, Q_d = 5 C·cm^{-2}) with pH = 4.8 after Cu_2O reduction.

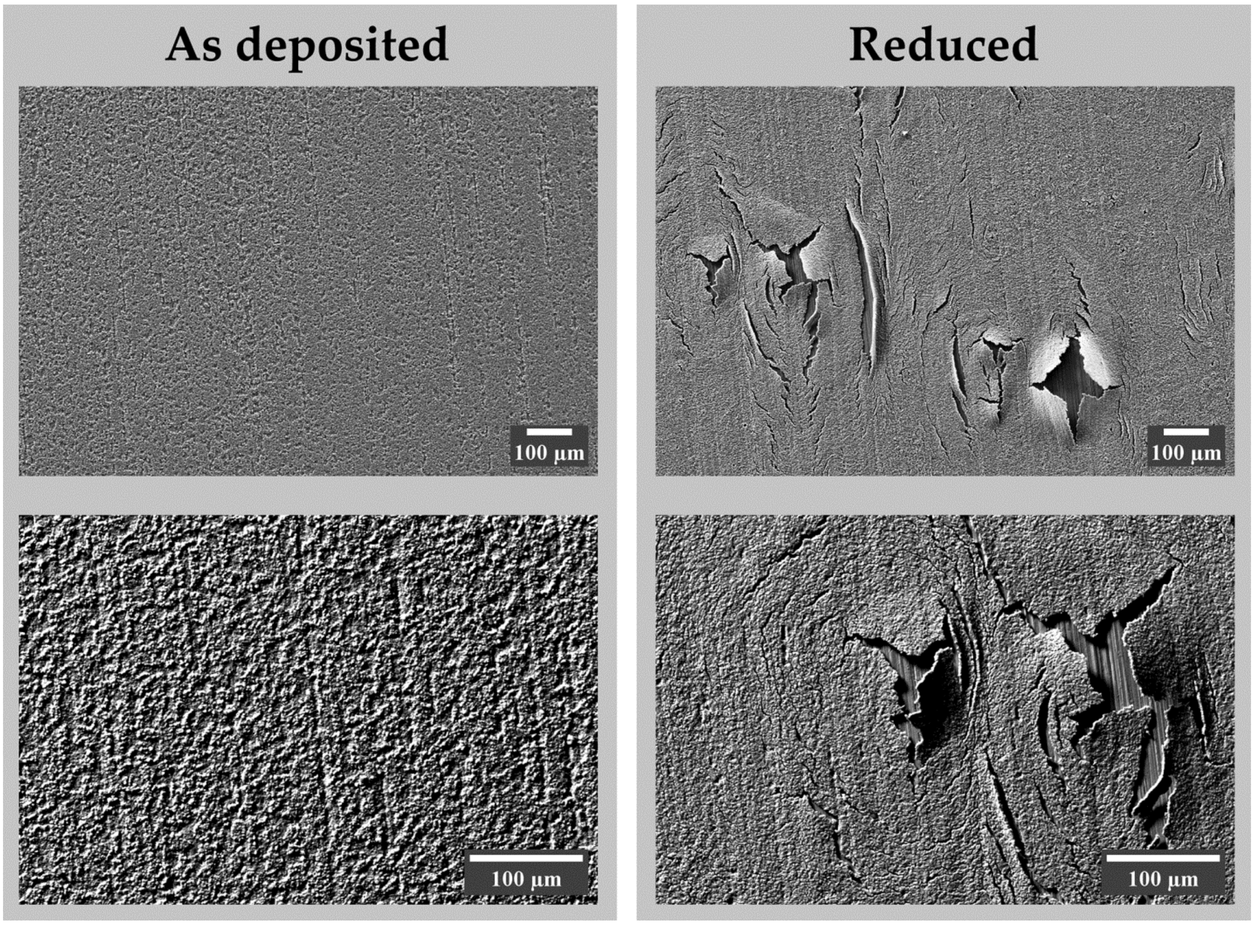

Figure S6. SEM images of the specimens deposited from lactate solution (E_d = –0.500 V, Q_d = 15 C·cm^{-2}) with pH = 7.9 after Cu_2O reduction.

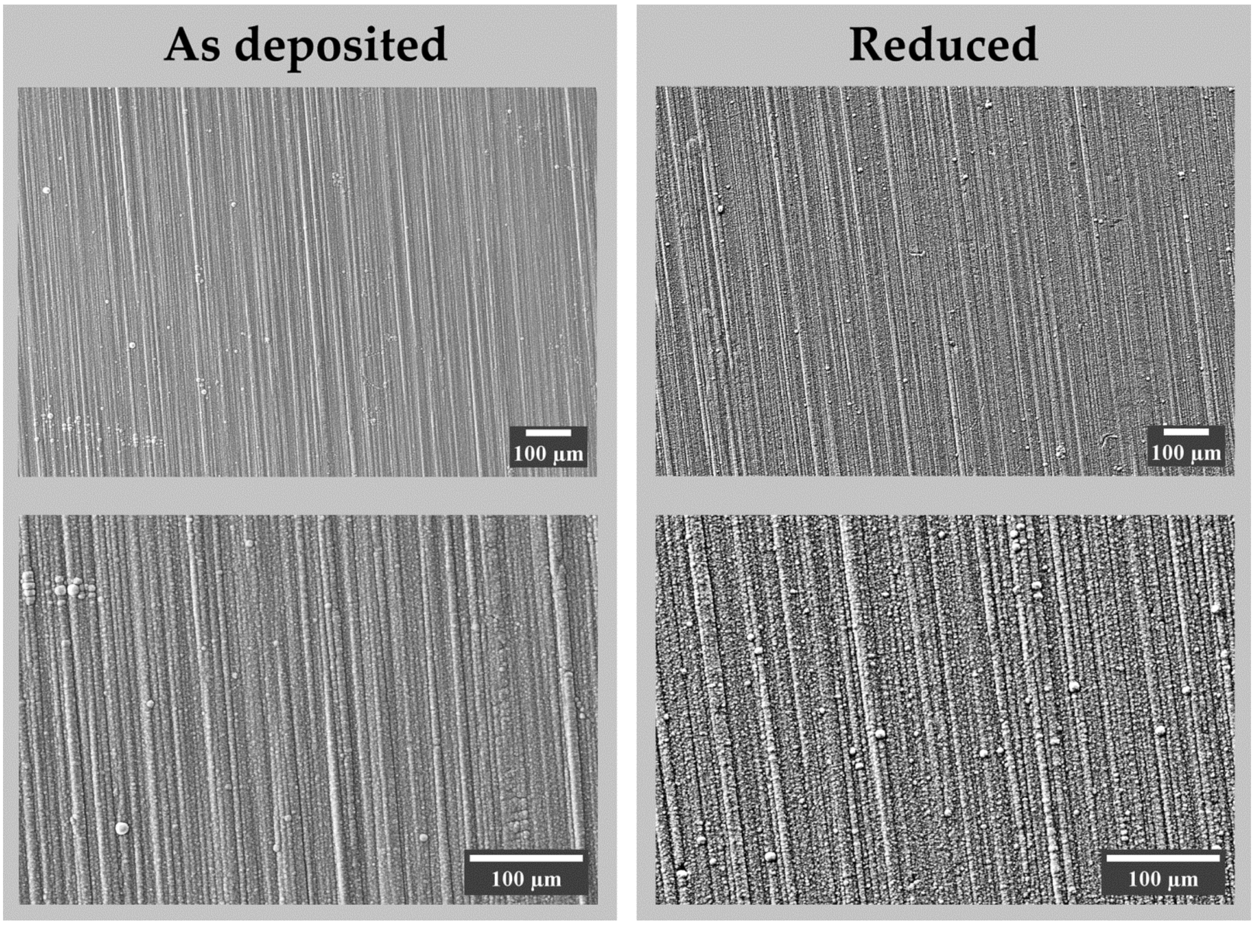

Supplement: Supplementary file 1 [file nanomaterials-13-03064-s001.zip › nanomaterials-2734105-supplementary.pdf]
